# Alterations of conjunctival microbiota associated with orthokeratology lens wearing in myopic children

**DOI:** 10.1186/s12866-023-03042-1

**Published:** 2023-12-13

**Authors:** Ju Zhang, Xiuhai Lu, Zhiwei Cheng, Dulei Zou, Weiyun Shi, Ting Wang

**Affiliations:** 1 Shandong First Medical University & Shandong Academy of Medical Sciences, Jinan, China; 2 Eye Hospital of Shandong First Medical University (Shandong Eye Hospital), 372 Jingsi Road, Jinan, 250021 China; 3 State Key Laboratory Cultivation Base, Shandong Provincial Key Laboratory of Ophthalmology, Shandong Eye Institute, Qingdao, China; 4 Medical College, Qingdao University, Qingdao, China

**Keywords:** Orthokeratology lenses, Conjunctiva sac, Microbiome, *Brevundimonas*, *Acinetobacter*, *Proteus*, Genomics, 16S rDNA gene sequencing

## Abstract

**Background:**

Orthokeratology (OK) lens wear increases the risk of bacterial infection, but little is known about the microbiota of the conjunctival sac in myopic children wearing OK lenses. This study aimed to investigate the changes of conjunctival microbiota in children after treatment with OK lenses using 16 S rDNA sequencing.

**Methods:**

Twenty-eight myopic children who had been continuously wearing OK lenses for 12 to 13 months were enrolled in this prospective study. Twenty-two gender- and age-matched myopic children who had not worn OK lenses or discontinued OK lens wear at least 1 year ago were recruited as controls. Conjunctival swabs from each participant were collected for exploration of the microbiota profiles, targeting the V3–V4 regions of the 16 S rRNA gene by MiSeq sequencing. The differences in the microbial community structure and diversity were also compared between groups.

**Results:**

The bacterial alpha diversity indices in the OK lens group were not different from those in the non-wearer group (*P* > 0.05, Wilcoxon test), while beta diversity examined using principle coordinate analysis of unweighted UniFrac divided the two groups into different clusters. *Proteobacteria*, *Bacteroidetes*, and *Firmicutes* were the abundant phyla in the conjunctival sac microbiota in both groups (*P* < 0.05, Mann–Whitney U test). Among children in the OK lens group, the Linear discriminant analysis Effect Size identified the compositional changes in OK lens-associated bacteria. Key functional genera such as *Blautia*, *Parasutterella*, and *Muribaculum* were enriched, whereas *Brevundimonas*, *Acinetobacter*, *Proteus*, and *Agathobacter* decreased significantly (*P* < 0.05, Mann–Whitney U test). Phylogenetic investigation of communities by reconstruction of unobserved states also showed altered bacterial metabolic pathways in OK lens-associated microbiota. Moreover, using receiver operating characteristic curves, *Brevundimonas*, *Acinetobacter*, *Proteus*, and *Agathobacter* alone (the area under the curve was all > 0.7500) or in combination (the area under the curve was 0.9058) were revealed to discriminate OK lens wearers from controls.

**Conclusions:**

The relative abundance of the microbial community in the conjunctival sac of myopic children can alter after OK lens wear. *Brevundimonas*, *Acinetobacter*, *Proteus*, and *Agathobacter* may be candidate biomarkers to distinguish between OK lens wearers and non-wearers.

**Supplementary Information:**

The online version contains supplementary material available at 10.1186/s12866-023-03042-1.

## Background

Myopia is predicted to affect half of the world’s population and become one of the leading causes of irreversible blindness by 2050 [1]. Orthokeratology (OK) is the use of rigid gas-permeable contact lenses with an inverse geometric design which can temporarily flatten the central corneal curvature to reduce the degree of myopia and improve uncorrected visual acuity (UCVA) in children and adolescents [2–5]. The growing incidence of myopia in the young population enables OK lenses increasingly used nowadays [6–8]. The sale volume of OK lenses in China was about 3 million pairs in 2020. However, OK lens wear enhances the risk of bacterial infection because lenses directly touch the surface of the cornea and are worn overnight [9]. Moreover, wearing contact lenses changes the microbiota of the conjunctival sac, which has been identified as a risk factor of ocular infections, like keratitis and macropapillary conjunctivitis, and corneal infiltrative events [10–12]. Recent studies have shown that the incidence of bacterial keratitis, one of the vision-threatening complications, [13–16]. was 13.9/10,000 in adolescents wearing OK lenses [17, 18]. Daily and extended contact lens wear has been found to cause an increase of *Staphylococci*, mainly *S. epidermidis* [19] and *S. aureus* [20]. Contact lens wearers might have more variable and skin-like bacterial community structures, with higher abundance of *Methylobacterium*, *Lactobacillus*, *Acinetobacter*, and *Pseudomonas* [10]. Zhang et al. [21] found that the abundance of *Bacillus, Tatumella*, and *Lactobacillus* was less in OK lens wearers than in non-wearers. These changes in the ocular microbiome have been suggested to affect the infection development in individuals wearing contact lenses. Nonetheless, the effect of contact lens wear on ocular microbiota has been inconclusive. It is necessary to further elucidate the interplay between microbiome and contact lens usage for better control of the complications [22, 23]

Although previous studies using the conventional culture technique have proved that applications of soft contact lenses can cause bacterial contamination, [19, 24] little is known about the impact of OK lenses on the structure and function of the microbiota on the surface of eyes, especially the microbiota of the conjunctival sac in children wearing OK lenses. In this study, 16 S rDNA gene high-throughput sequencing-based bacterial detection and identification [25] were performed, aiming to explore the true diversity of conjunctival microbiota in pediatric OK lens wearers and thus to guide clinical management of associated ocular surface inflammation.

## Subjects and methods

### Subjects

Myopic patients who had been wearing OK lenses for 12–13 months and myopic patients who had not worn OK lenses or discontinued OK lens wear at least 1 year ago, aged 8–15 years, were prospectively enrolled at the Eye Hospital of Shandong First Medical University from September 2020 to December 2020. Among the participants, there were 14 males and 14 females in the OK lens group (L group), averaged 12.5 ± 2.6 years old, and 11 males and 11 females in the non-wearer group (N group), averaged 10.0 ± 2.2 years old. No significant differences in gender (*P* > 0.05, Chi-square test) and age (*P* > 0.05, Student’s T-test) existed between the two groups. Patients who had a history of eye surgery or trauma, other ocular or systemic diseases, antibiotic, anti-inflammatory or immunosuppressive medications within 6 months were excluded from the study.

**Fig. 1:**
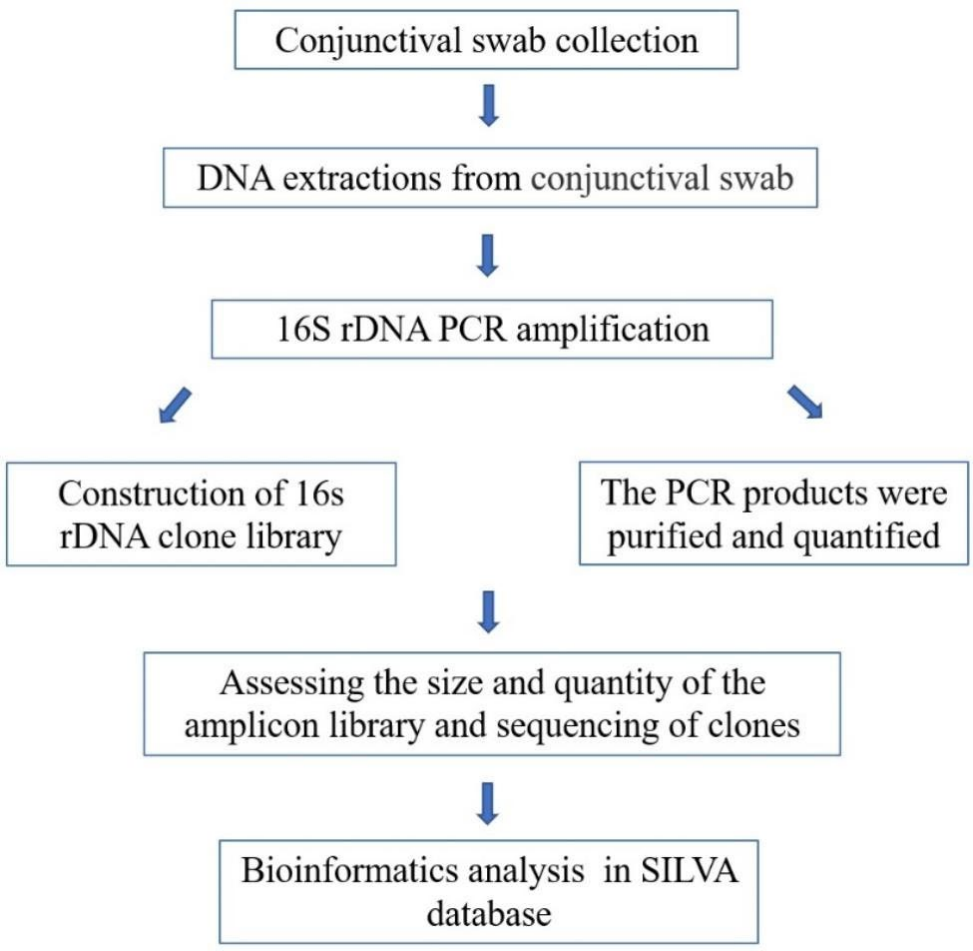
A schematic diagram of the research procedure beginning from conjunctival swab collection, followed by DNA extractions, PCR amplification, construction of 16s rDNA clone library, sequencing of clones, and bioinformatics analysis

This study was approved by the Ethics Committee of the Eye Hospital of Shandong First Medical University (SDSYKYY20200713) and registered on the Chinese Clinical Trial Registry (ChiCTR2000037230, 27/08/2020). All the procedures adhered to the tenets of Declaration of Helsinki. Written informed consent was obtained from legal guardians of each participant. A schematic diagram of the research procedure is shown in Fig. 1.

### Conjunctival swab collection

Conjunctival swab samples were taken from the right eye of each patient by the same clinician to ensure consistency. To eliminate the influence of anesthetics on the conjunctival sac microbiota, anesthetic eye drops were not used in the sample collection process [10]. The lower palpebral and fornix conjunctiva was swabbed three times using a sterile cotton swab (CLASSIQSwabs; Copan, Brescia, Italy), which avoided any contact with the eyelid margin and was rotated toward the opposite direction of the conjunctiva to maximize effective collection. Then the samples were immediately placed in a sterile, RNase-free cryopreservation tube, frozen with liquid nitrogen, and preserved in a refrigerator at minus 80 ℃. 16 S rDNA gene high-throughput sequencing was performed within 1 month after sample collection.

### DNA extraction

According to the instructions of the PowerMax® Soil DNA Isolation Kit, the DNA extraction of all the samples was completed in a biosafety cabinet. The DNA purity and concentration were checked by ultraviolet spectrophotometer reading. The quality of the extracted DNA was verified using agarose gel electrophoresis, and the quantification of DNA was achieved by ultraviolet spectrophotometry. Throughout the DNA extraction process, ultrapure water instead of a sample solution was used to exclude the possibility of false-positive polymerase chain reaction (PCR) results.

### PCR amplification and 16 S rDNA sequencing

The primers were designed according to the conserved regions in the ribosomal RNA of microorganisms as 341F (5’-CCTACGGGNGGCWGCAG-3’) and 805R (5’-GACTACHVGGGTATCTAATCC-3’), and universal adapters and barcode sequences were added for PCR amplification of the V3-V4 variable regions [26]. The 5’ ends of the primers were tagged with specific barcodes per sample and sequencing universal primers. PCR amplification was performed in a 25-µL reaction mixture containing 25 ng of template DNA, 12.5 µL of PCR premix, 2.5 µL of each primer, and PCR-grade water to adjust the volume. The PCR conditions to amplify the prokaryotic 16 S fragments consisted of an initial denaturation at 98 ℃ for 30 s, 32 cycles of denaturation at 98 ℃ for 10 s, annealing at 54 ℃ for 30 s, an extension at 72 ℃ for 45 s, and a final extension at 72 ℃ for 10 min [27]. The PCR products were purified using AMPure XT beads (Beckman Coulter Genomics, Danvers, MA, USA) and quantified using the Qubit fluorometer (Invitrogen, Carlsbad, CA, USA). The amplicon pools were prepared for sequencing, and the size and quantity of the amplicon library were assessed on the Agilent 2100 Bioanalyzer (Agilent, CA, USA) and with the Library Quantification Kit for Illumina (Kapa Biosciences, Woburn, MA, USA), respectively. The libraries were sequenced on the NovaSeq PE250 platform.

### Bioinformatics analysis

Samples were sequenced on an Illumina NovaSeq platform according to the manufacturer’s recommendations at the LC-Bio (Hangzhou, China). After dereplication using Divisive Amplicon Denoising Algorithm 2, [28] we obtained the feature tables and feature sequences. Alpha diversity and beta diversity were calculated by random normalization to the same sequences. Alpha diversity was applied in analyzing complexity of species diversity for each sample with indices of Chao1, Observed species, Goods coverage, Shannon, and Simpson calculated using quantitative insights into microbial ecology 2 [29]. Beta diversity calculated using principle coordinate analysis derived from unweighted UniFrac distances was used to indicate the difference in the overall composition and distribution of the microbial community between groups [30]. Blast was used for sequence alignment, and the feature sequences were annotated with the SILVA database for each representative sequence.

### Statistical analysis

All data were statistically analyzed by SPSS20.0 software. The t-test and Mann-Whitney U test were applied for continuous variables. The Chi-square was used for categorical variables between groups. The R package (v3.5.2) was used for preparation of graphs. All tests of significance were two sided, and *P* < 0.05 or corrected *P* < 0.05 was considered statistically significant. The sample size was determined from G*Power [31].

### Accession number

The sequence data from this study are deposited in the GenBank Sequence Read Archive with the accession number PRJNA819236.

## Results

### Changed overall structure of conjunctival microbiota in children wearing OK lenses

**Fig. 2:**
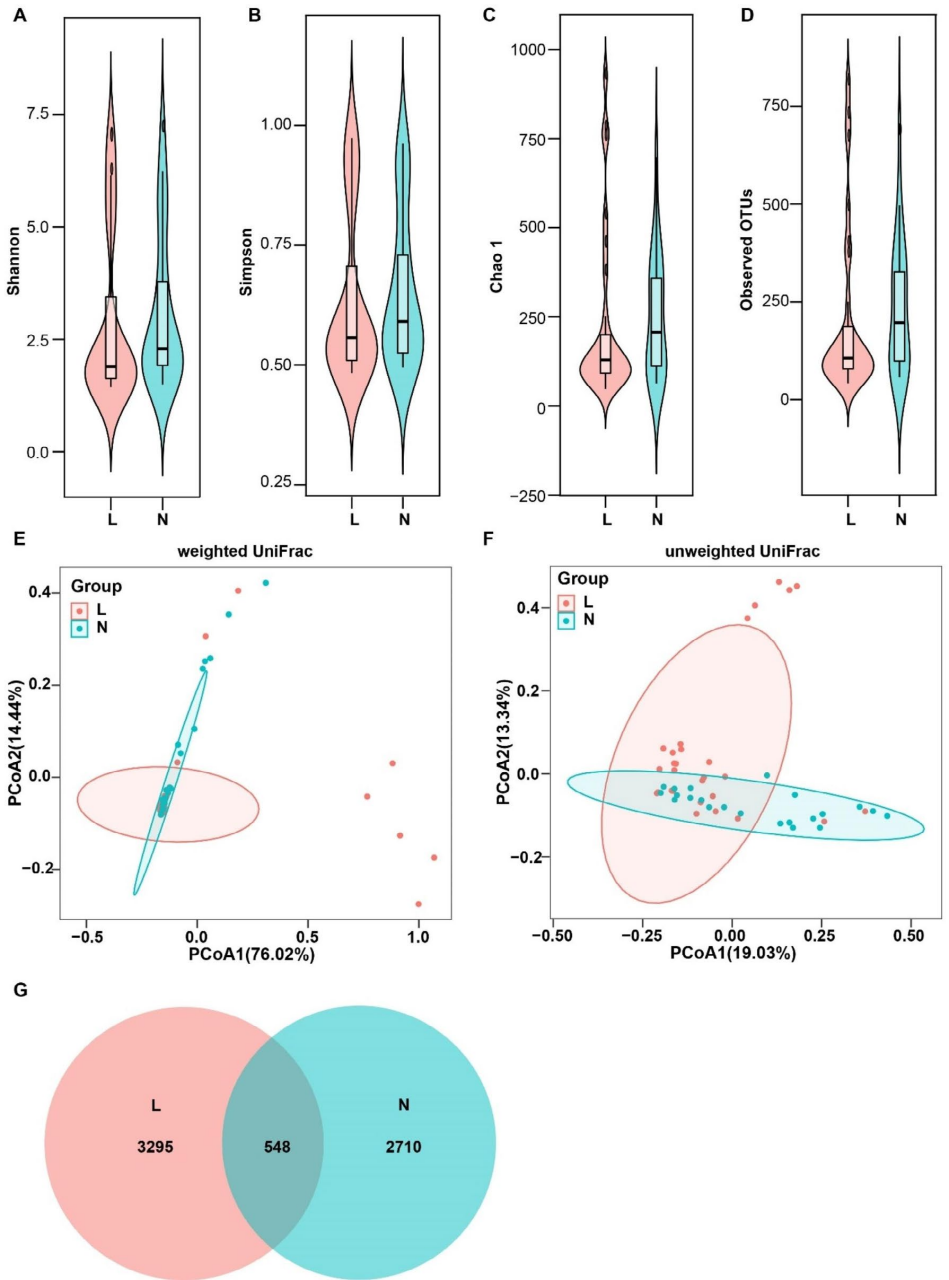
The bacterial diversity and richness of the conjunctival sac microbiota in two groups. **A-D**: Violin plots representing alpha diversity of different individuals in the two groups (*P >* 0.05, Mann-Whitney U test). **E-F**: Principal coordinates analysis plots. Each point represents a sample, and the points of the same color are from the same group. The closer the distance between two points, the smaller the difference in community composition between the two groups (*P*<0.05, ANOSIM test). **G**: The Venn diagram illustrates the overlap of OTUs in the conjunctival microbiota among the two microhabitats. L: the OK lens group, N: the non-wearer group

In total, 3,920,380 high-quality reads (2,187,649 for the OK lens group and 1,732,731 for the non-wearer group), with an average of 78,407 reads per sample, were obtained for the subsequent microbiota analysis. The value of Good’s coverage was 99.83%, indicating that a majority of bacterial phylotypes [7,177 operational taxonomic units (OTUs)] in the conjunctival microbiota were identified. It was observed that bacterial alpha diversity indices of Shannon, Simpson, Chao1, and observed OTUs were not significantly different between groups (Wilcoxon test, *P* > 0.05; Fig. 2A-D). The rarefaction curve of each sample tended to be flat, indicating that most of the bacteria were detected (supplementary 1). Beta diversity also divided the two groups into different clusters (ANOSIM test, *P* < 0.05; Fig. 2E and F). The Venn diagram illustrates the overlap of OTUs in the conjunctival microbiota of the two microhabitats (Fig. 2G).

### Altered conjunctival microbiota composition in children wearing OK lenses

**Fig. 3:**
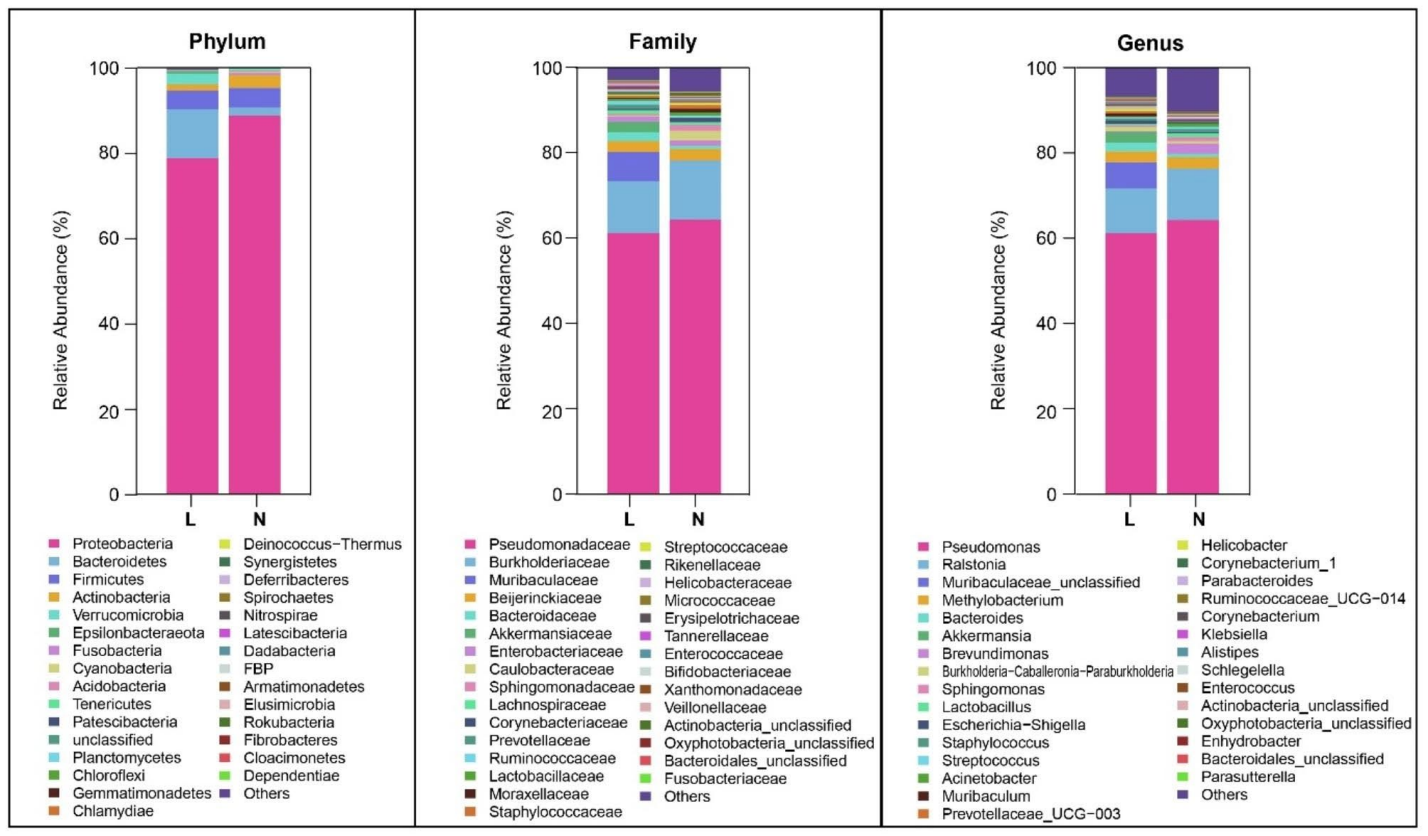
Histogram of bacterial taxa distribution at the phylum, family, and genus levels in the two groups. The top 30 species with the highest relative abundance in the two groups. The vertical axis represents the relative abundance of each species, whereas the horizontal axis is the group name. The columns of different colors correspond to different bacteria, and the length of the columns represents the proportion of the bacterial taxa. L: the OK lens group, N: the non-wearer group

**Fig. 4:**
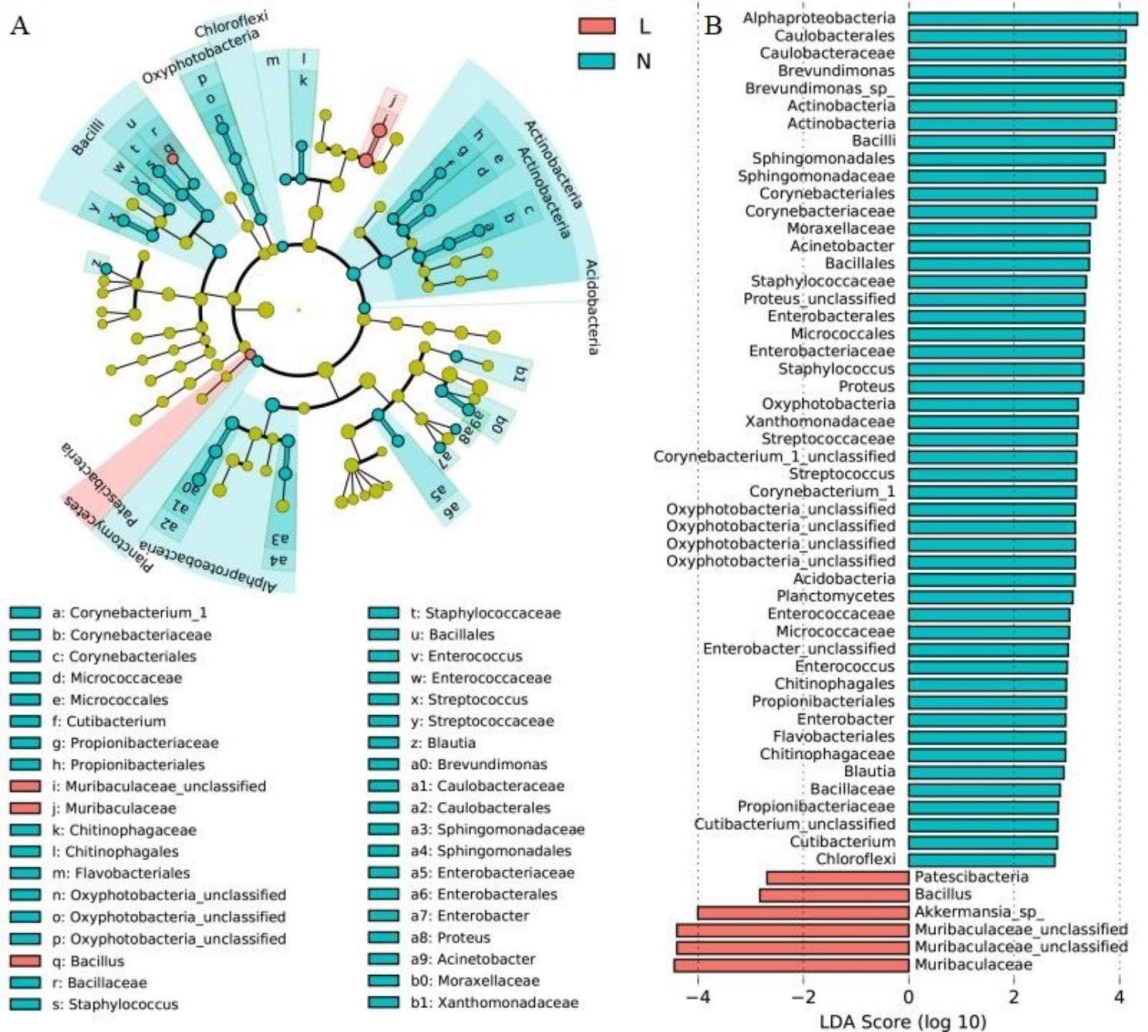
Linear Discriminant Analysis (LDA) Effect Size (LEfSe) multilevel discriminant analysis of the species differences (LDA > 3, *P* < 0.05, Mann–Whitney U-tests). A: Cladogram. The circle radiating from inside to outside represents the classification level from the kingdom to the genus (or species). Species with no significant differences are uniformly colored in yellow, the red nodes represent the microbial group that plays an important role in the OK group, and the green nodes represent the non-wearer group. B: Histogram of LDA value distribution shows the biomarkers with statistical difference. The LDA value represents the influence of bacterial species, and the longer the length, the higher the degree of influence. L: the OK lens group, N: the non-wearer group

**Fig. 5:**
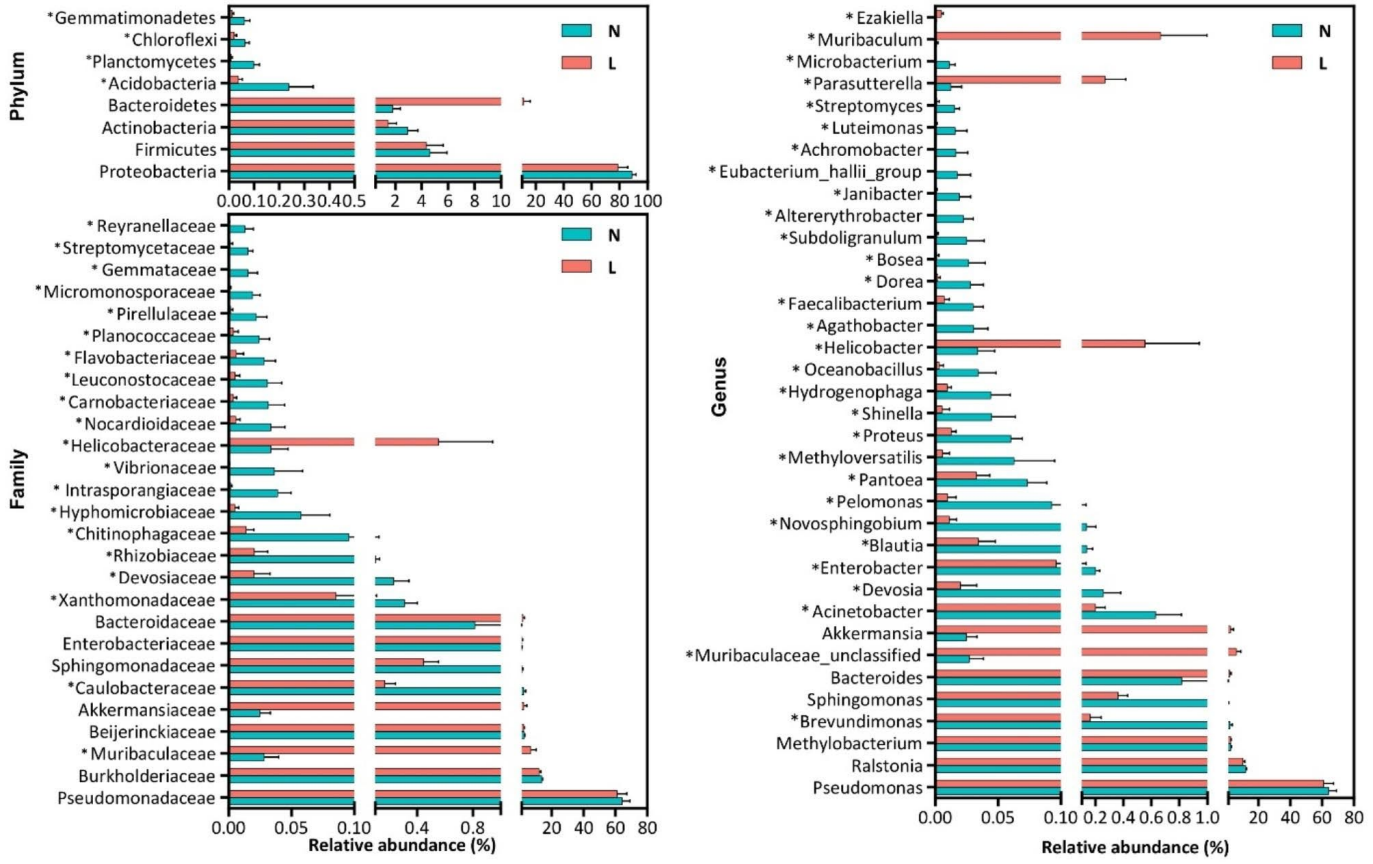
Comparisons of the relative abundance of the bacterial taxa at the phylum, family, and genus levels. The data are presented as the mean ± standard error. The Mann–Whitney U test is used to analyze variation between the OK lens wearers and the non-wearers. *P < 0.05 compared with the non-wearer group. L: the OK lens group, N: the non-wearer group

The compositions of conjunctival microbiota in the OK lens wearers and the controls were assessed at different taxonomic levels. Using the Ribosomal Database Project (RDP) classifier, the sequences were classified into 37 phyla, 304 families, and 778 genera. At the phylum level, the abundance of *Actinobacteria* was significantly reduced in the OK lens group (1.47% vs. 2.95%, *P* = 0.00, Wilcoxon test). The abundant bacterial genera in both groups were *Pseudomonas* (61.18% vs. 64.26%, *P* = 0.56, Wilcoxon test), *Ralstonia* (10.48% vs. 12.04%, *P* = 0.92, Wilcoxon test), and Muribaculaceae-unclassified (6.18% vs. 0.03%, *P* = 0.00, Wilcoxon test). The abundance of Muribaculaceae-unclassified was significantly increased in the OK lens group (Fig. 3). In terms of bacterial phenotypes, the abundance of facultative anaerobe decreased significantly in the OK lens group (5.07% vs. 8.23%, *P* = 0.009, Wilcoxon test; supplementary 2). The Linear discriminant analysis (LDA) Effect Size (LEfSe) identified the differential bacteria at different taxonomic levels between the two groups (LDA score > 3, *P* < 0.05, Mann-Whitney U test). Among the key functional bacteria identified in the conjunctival microbiota, *Muribaculaceae-unclassified*, *Blautia*, *parasutterella*, and *Muribaculum* had higher abundance at the genus level in the OK lens group, while the abundance of *Brevundimonas*, *Acinetobacter*, *Proteus*, and *Agathobacter* was higher in the non-wearer group (Figs. 4 and 5). These differential genera were candidate biomarkers to discriminate the two groups of patients.

### Discrimination with conjunctival microbiota-based signatures

**Fig. 6:**
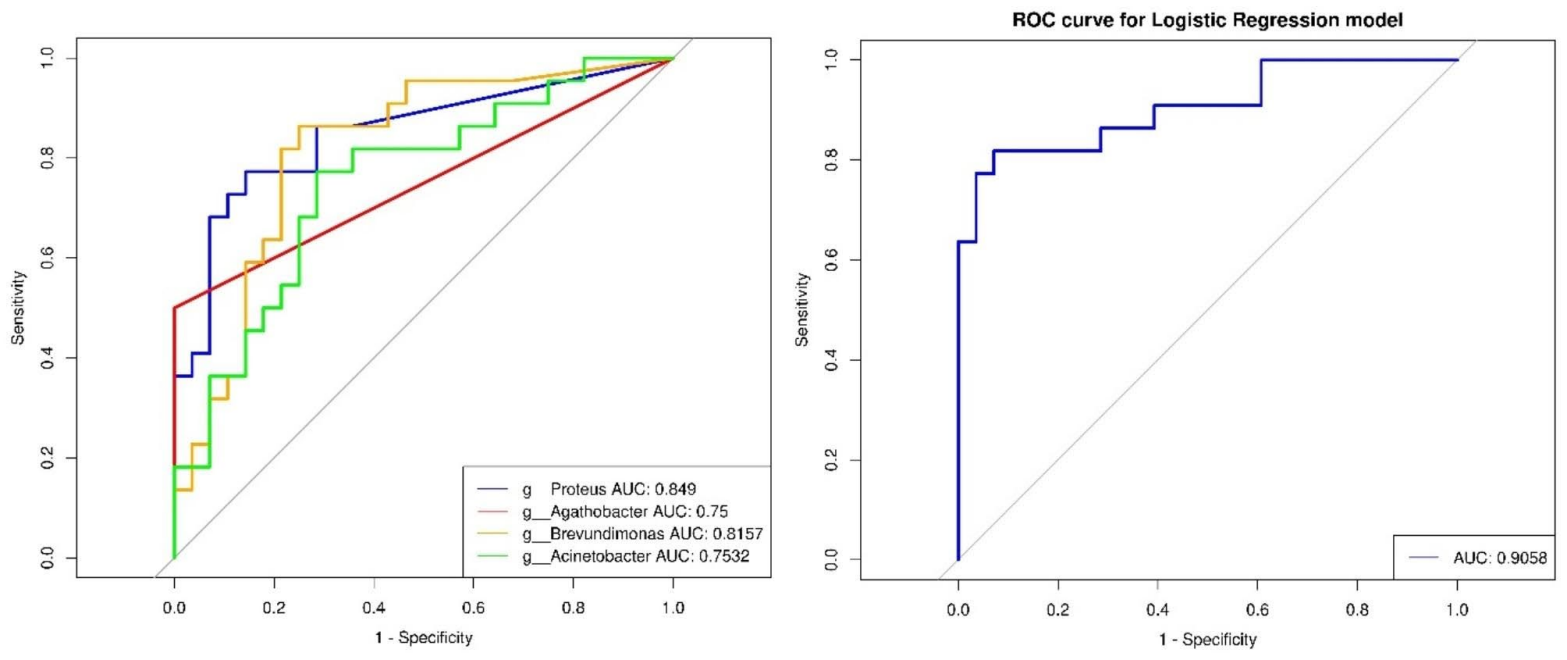
The differential microbiota as diagnostic markers of the OK lens group. Receiver operating characteristic (ROC) curves for the differential microbiota alone or in combination are used to discriminate the OK lens wearers from the non-wearers. AUC, the area under the receiver-operating characteristic curve. L: the OK lens group, N: the non-wearer group

We used each single differential bacterial genus as predictor to generate the area under the receiver-operating characteristic (ROC) curves (AUC) and observed the values of four abundant genera as biomarkers: *Proteus* (AUC = 0.8490), *Brevundimonas* (AUC = 0.8157), *Acinetobacter* (AUC = 0.7532), and *Agathobacter* (AUC = 0.7500). We also applied multivariable stepwise logistic regression analysis to further distinguish the OK lens wearers from the non-wearers, using the four abundant genera. The predictive performance was significantly improved (AUC = 0.9058) (Fig. 6).

### Microbial functional dysbiosis in OK lens wearers

**Fig. 7:**
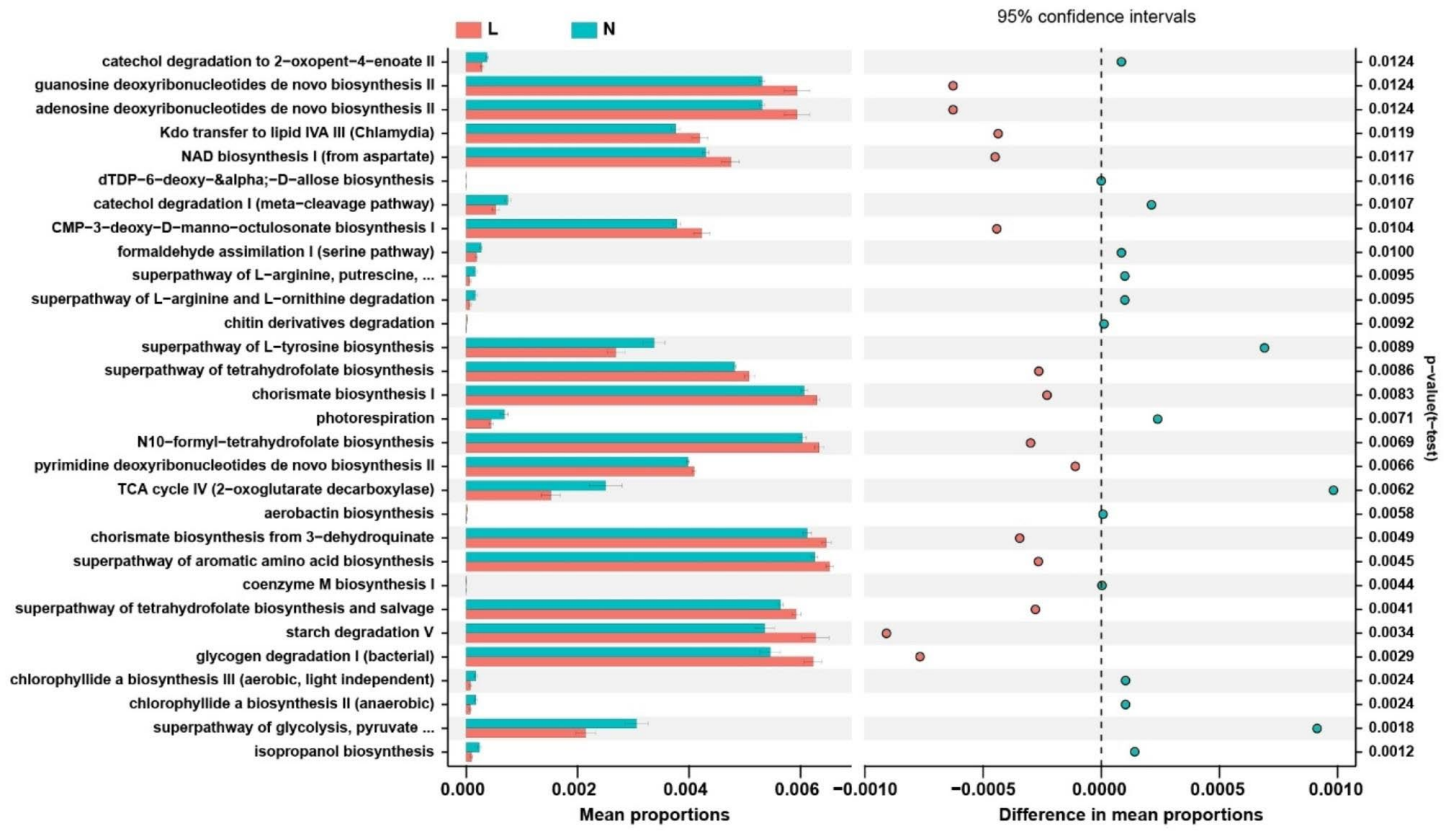
PiCRUSt-based examination of the conjunctival microbiome of the OK lens wearers and the non-wearers. The different bacterial functions are evaluated between them based on the t-test. Comparisons between the groups for each KEGG functional category are shown by percentage

To identify the metabolic and functional disparities in the conjunctival microbiota between the OK lens wearers and the non-wearers, PiCRUSt was used to analyze the functional potential of the microbiota based on closed-reference OTU picking. We compared 64 KEGG pathways and identified several KEGG categories with clearly differential abundance between the two groups, finding that carbohydrate metabolism, xenobiotics’ biodegradation and metabolism, and transport and catabolism significantly increased after OK lens wear, while transcription, immune system, and environmental adaptation significantly decreased (*P* < 0.05, t- test; Fig. 7).

## Discussion

Contact lens wear is a known risk factor that may cause microbial keratitis and other ocular inflammation [10, 32–37]. Previous studies have disclosed that the microbiota of the conjunctival sac has a protective effect against foreign bacterial invasion [38]. However, wearing contact lenses can alter the microbiome of the ocular surface, which may reduce the restoration of the conjunctival microenvironment [10, 39, 40]. Moreover, the positive detection rate of the conventional bacterial culture method was reported to be only 34-65% [41–44]. Such culture results are not efficient to reflect the microenvironment status of the bacterial community in the conjunctival sac [11, 45]. In this study, we comprehensively examined changes in the microbial community structure of the conjunctival sac in children wearing OK lenses. Using 16 S rDNA gene sequencing can accurately detect and identify slowly growing, non-culturable or culture-resistant bacteria and those with special growth requirements, such as *Pseudomonas*, *Acinetobacter*, *Brevundomonas*, *Sphingomonas*, and *Streptococcus*, [10, 25] besides bacteria which have been detectable using the conventional bacterial culture, like *coagulase-negative Staphylococcus*, *Propionibacterium*, and *Corynebacterium* [46].

Graham et al. [25] measured the conjunctival sac microbiome of healthy people by 16 S rDNA sequencing, finding the high abundance of bacteria belonged to *S. epidermidis*, *Coagulase-negative Staphylococcus*, *Corynebacterium sp.*, and *P. acnes.* Dong et al. [39] identified the most abundant bacterial genera in the conjunctival microbiota, namely *Pseudomonas*, *Bradyrhizobium*, *Propionibacterium*, *Acinetobacter*, and *Corynebacterium*. In our series study, the top five bacterial genera with high abundance in the conjunctival sac of children, no matter with or without OK lens wear, included *Pseudomonas*, *Ralstonia*, *Muribaculaceae-unclassified*, *Methylobacterium*, and *Bacteroides*. The inconsistency in the predominant species, except *Pseudomonas*, might be due to the age and region differences of the subjects [47–49]. In addition, the possibility that the location and depth of sampling may affect the result cannot be entirely excluded [50, 51]. The current study supports that *Pseudomonas* might be one of the colonizing bacteria in the conjunctival sac, [52] which is different from the traditional culture results [53]. However, once the epithelial barrier of the OK lens wearers is destroyed, [54]*Pseudomonas* can rapidly pass through the barrier, bind to the surface, and cause bacterial aggregates and membrane remodeling. The process may evade the immune surveillance and result in ocular infections [55]. This is consistent with the rapid and violent course of *Pseudomonas aeruginosa* infection in OK lens wearers.

In our study, the number of bacterial species in the OK lens group was reduced compared with that in the non-wearer group, which may be attributed to the following reasons. On one hand, the bacterial residues due to inadequate contact lens care may lead to changes and establish the indigenous microbiome in the conjunctival sac. On the other hand, the increase in the abundance of certain pathogenic bacteria may inhibit other bacteria. Regarding the abundance distribution of some bacteria, the relative abundance of *Brevundimonas*, *Acinetobacter*, *Proteus*, and *Agathobacter* in the OK lens group decreased, while that of *Muribaculaceae-unclassified*, *Blautia*, *Parasutterella*, and *Muribaculum* increased, breaking the balance of the indigenous microbial environment. Accumulating evidence disclosed that the opportunistic pathogens, such as *Brevundimonas*, *Proteus*, and *Acinetobacter*, increased the potential risk of ocular surface infection [56–58]. Wang et al. [59] discovered that *Blautia* producta was positively correlated with the disease course of lens-associated *Acanthamoeba* keratitis. *Acanthamoeba* is one of the most common etiological agents of infectious keratitis in association with OK lens wear [14]. The intervention of *Blautia* flora seems to be promising for the treatment of lens-associated *Acanthamoeba* keratitis. Yun et al. [60] found that *Actinobacteria* populations at the phylum level and *Muribaculaceae* at the family level of gut bacteria were clearly related to the secretion of tears. We believe that the decreased tear secretion at night after wearing OK lenses may reduce the washing effect of the tear film on bacteria and increase the risk of infection. Meanwhile, it was revealed that the abundance of facultative anaerobe in the OK lens group was significantly lower than that in the non-wearer group. Wearing OK lenses at night may lead to different degrees of hypoxia in the conjunctival sac, [61] and facultative anaerobes are capable of switching to fermentation or anaerobic respiration but do not reproduce. Therefore, there was a decrease of abundance, which was also an influencing factor of the microbial abundance in the conjunctival sac.

This study demonstrated that the abundance of some bacterial populations changed obviously after OK lens wear at the levels of phylum and genus. For example, at the genus level, *Proteus*, *Agathobacter*, *Brevundimonas*, and *Acinetobacter* reduced markedly, so they were selected alone or together for the ROC analysis as non-invasive biomarkers to distinguish the OK lens wearers from the non-wearers. This provides a new target for early warning and intervention of OK lens-related flora alterations and possible risks.

Ozkan et al. [53] analyzed the microbial community in the conjunctival sac of healthy people by culture, disclosing that Gram-positive microorganisms accounted for the majority of the isolated microorganisms (94%), and the most frequently isolated genus was *Staphylococcus* (46.5%), which was not very abundant in gene sequencing in our study. On the contrary, *Pseudomonas* had high gene sequencing abundance, but its positive rate in the traditional culture was low. It seems that a reasonable combination of the two detection methods can better serve clinical needs.

Considering when people put contact lenses into eyes with their fingers, they may touch the eyelashes and eyelids, Costello et al. [62] regarded contact lenses as a medium that can transmit bacteria from the skin of hands or eyelids into the eyes and result in microbial imbalance. Therefore, the factor of daily care should also be involved in the changes of conjunctival sac floras after wearing OK lenses. Thorough and careful hand washing before each insertion and removal of the contact lenses and less contact with the skin are highly recommended.

Further studies are required to overcome the limitations in this research. First, the host response related to the floras should be investigated to better reflect the association with the disease. Second, more samples collected from a wider range of subjects at different time points are necessary. Third, since a large number of sequences remain unknown, it is difficult to identify strains to the species level using 16 S rDNA sequencing, which is needed to be improved. [63].

## Conclusion

In conclusion, using 16 S rDNA gene sequencing, this study confirms that the relative abundance of bacterial taxa can change after OK lens wear in myopic children, affecting the physiological and immune status of the ocular surface. *Brevundimonas, Acinetobacter, Proteus*, and *Agathobacter* may be candidate biomarkers to distinguish between OK lens wearers and non-wearers. The findings are conducive to understanding the role of ocular surface microbiota in the ocular surface inflammation in pediatric OK lens wearers.

### Electronic supplementary material

Below is the link to the electronic supplementary material.

Supplementary Material 1

## Data Availability

The data analyzed in the current study have been uploaded to https://www.ncbi.nlm.nih.gov/bioproject/819236 and the Sequence Read Archive (SRA) (accession number PRJNA819236).

## Abbreviations

Orthokeratology: OK
LDA: Linear Discriminant Analysis
LEfSe: Linear discriminant analysis effect size
PICRUSt: Phylogenetic investigation of communities by reconstruction of unobserved states
ROC: Receiver-operating characteristic
AUC: Area under the receiving-operating characteristic curves
UCVA: uncorrected visual acuity
OUT: Operational taxonomic unit
RDP: Ribosomal Database Project
