# Supplementary material for: Alterations of conjunctival microbiota associated with orthokeratology lens wearing in myopic children
Source: BMC Microbiol. 2023 Dec 13;23:397. doi: 10.1186/s12866-023-03042-1 (PMC10717905; doi:10.1186/s12866-023-03042-1)
Supplement: Supplementary file 1 — Supplementary Material 1 [file 12866_2023_3042_MOESM1_ESM.docx]

Supplement 1.


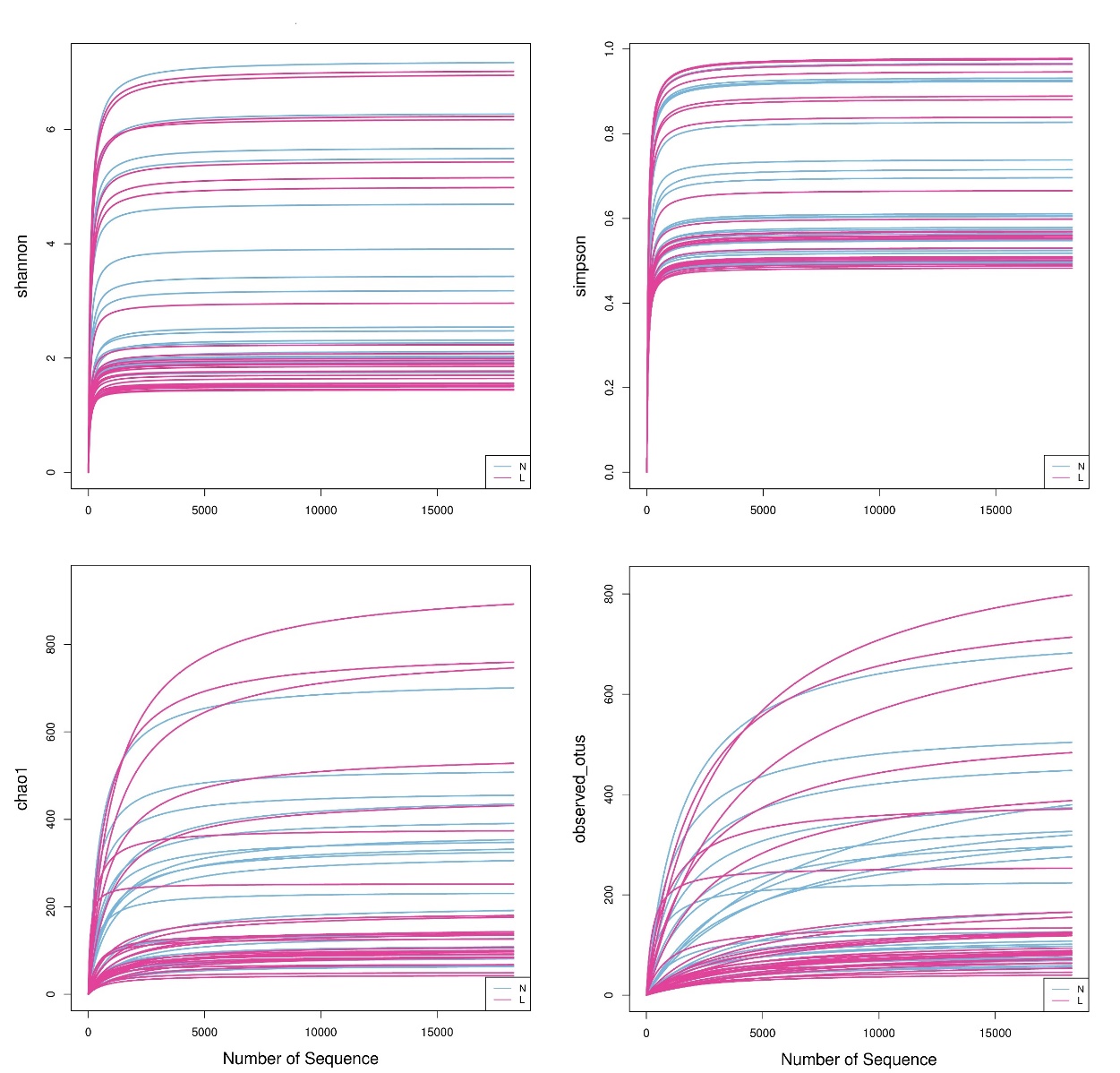


The rarefaction curve of each sample tended to be flat, indicating that most of the bacteria were detected.

Supplement 2.


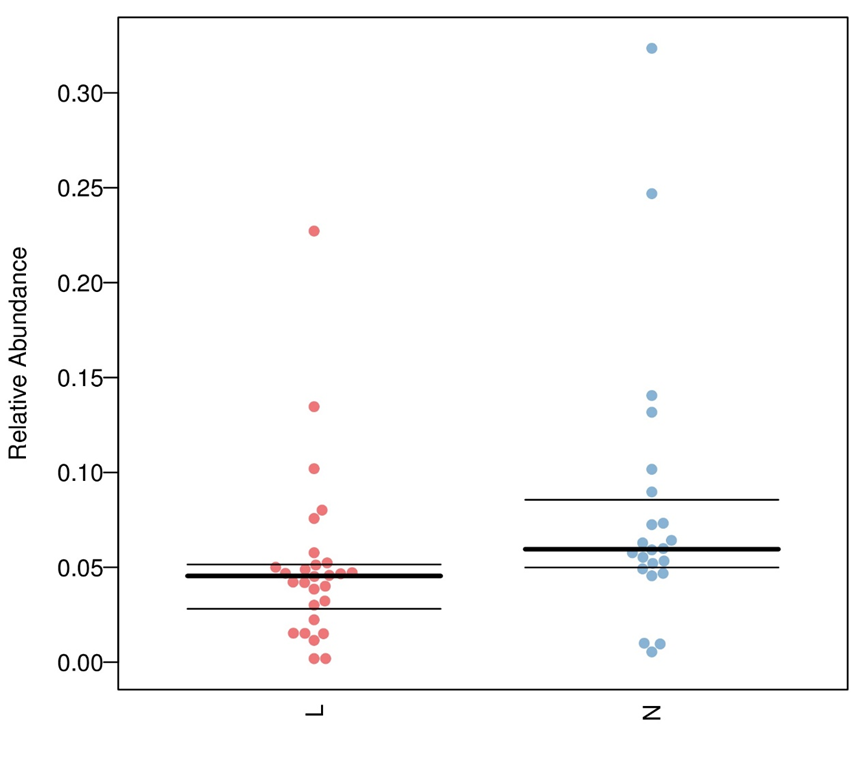
 In terms of bacterial phenotypes, the abundance of facultative anaerobe decreased significantly in the OK lens group (5.07% vs. 8.23%, P = 0.009).
